# CD 36: Focus on Epigenetic and Post-Transcriptional Regulation

**DOI:** 10.3389/fgene.2019.00680

**Published:** 2019-07-19

**Authors:** Cristina-Mariana Niculite, Ana-Maria Enciu, Mihail Eugen Hinescu

**Affiliations:** ^1^Cell Biology Department, “Victor Babes” National Institute of Pathology, Bucharest, Romania; ^2^Department of Cellular and Molecular Biology and Histology, “Carol Davila” University of Medicine and Pharmacy, Bucharest, Romania

**Keywords:** CD36, epigenetics, non-coding RNAs, inflammation, obesity, biomarkers

## Abstract

CD36 is a transmembrane protein involved in fatty acid translocation, scavenging for oxidized fatty acids acting as a receptor for adhesion molecules. It is expressed on macrophages, as well as other types of cells, such as endothelial and adipose cells. CD36 participates in muscle lipid uptake, adipose energy storage, and gut fat absorption. Recently, several preclinical and clinical studies demonstrated that upregulation of CD36 is a prerequisite for tumor metastasis. Cancer metastasis-related research emerged much later and has been less investigated, though it is equally or even more important. CD36 protein expression can be modified by epigenetic changes and post-transcriptional interference from non-coding RNAs. Some data indicate modulation of CD36 expression in specific cell types by epigenetic changes *via* DNA methylation patterns or histone tails, or through miRNA interference, but this is largely unexplored. The few papers addressing this topic refer mostly to lipid metabolism-related pathologies, whereas in cancer research, data are even more scarce. The aim of this review was to summarize major epigenetic and post-transcriptional mechanisms that impact CD36 expression in relation to various pathologies while highlighting the areas in need of further exploration.

## Introduction

Very different pathologies, such as cardiovascular disease, malaria, and tumor metastasis, share a mechanism involving the membrane glycoprotein CD36. It was first identified as an adhesion protein, mediating collagen, fibronectin, thrombospondin (TSP) (Tandon et al., 1989; Silverstein and Febbraio, 2009), and *Plasmodium falciparum* binding (Barnwell et al., 1989). CD36 was later demonstrated to be a scavenger receptor (Febbraio et al., 2001; Silverstein and Febbraio, 2009), a fatty acid translocator for native and oxidized low-density lipoprotein (oxLDL) (Endemann et al., 1993), anionic phospholipids (Rigotti et al., 1995), and long-chain fatty acids (FAs) (Abumrad et al., 1993; Glatz and Luiken, 2017). These different functions are performed by different binding sites of the extracellular domain of the receptor (Asch et al., 1993; Jay and Hamilton, 2018), and the outcome of ligand binding is largely dependent on cell type. In muscle, adipose, and intestinal cells, CD36 participates mainly in lipid uptake (Smith et al., 2008; Tran et al., 2011).

In macrophages, depending on the environment, CD36 can act as a scavenger of oxLDL, forming foam cells (Park, 2014) as a pattern recognition receptor, in innate immunity (Thylur et al., 2017), or as a trigger of inflammatory responses (Stewart et al., 2010; Qin et al., 2017). In endothelial cells, it has mainly anti-angiogenic and pro-apoptotic effects *via* thrombospondin binding (Klenotic et al., 2013). In vascular smooth muscle cells, CD36 may contribute to generation of reactive oxygen species (ROS) and cellular proliferation (Li et al., 2010; Yue et al., 2019). In sensory cells, it can act as a taste (Laugerette et al., 2005; Keller et al., 2012; Pepino et al., 2012; Ozdener et al., 2014) or olfactory receptor (Oberland et al., 2015; Xavier et al., 2016).

CD36 is currently investigated as a potential therapeutic target in cardiovascular disease (Portal et al., 2016), metabolic syndrome, and obesity (Corpeleijn et al., 2008; Goyenechea et al., 2008). Several preclinical (Pascual et al., 2017; Ladanyi et al., 2018; Sp et al., 2018) and clinical studies (Liang et al., 2018; Pan et al., 2019) recently demonstrated that upregulation of CD36 could be a prerequisite for tumor metastasis (reviewed in Enciu et al., 2018), opening new avenues for anticancer therapies. Even though the localization and changes in expression of CD36 protein have been sufficiently addressed, less is known about how epigenetic and post-transcriptional regulation of CD36 influences CD36-related pathologies. A more detailed look into these less explored areas is necessary to highlight gaps in knowledge, as well as new possible therapeutic targets. Therefore, the aim of this review was to summarize major epigenetic and post-transcriptional mechanisms with impact on CD36 expression in relation to various pathologies. The blank spots on the CD36 map are also addressed.

### CD36 Gene Regulation and Gene-Related Pathologies

The CD36 gene is located on the long arm of chromosome 7 (7q21.11) (*Homo sapiens* Annotation Release 109, GRCh38.p12) and consists of 17 exons and 18 introns and can generate four protein isoforms *via* alternative splicing (https://www.ncbi.nlm.nih.gov/gene/948). Out of the four isoforms, three have at least one spliced exon (4, 6-7, or 8) (UniProtkB ID: P16671). At least 23 alternative transcripts are known for CD36, and six alternative first exons have been described, yielding different transcripts in different tissue types (Pietka et al., 2014; Mikkelsen et al., 2010). A STAT binding GAS element has also been identified in the CD36 gene promoter (Sp et al., 2018), and several papers have reported that CD36 expression is modulated by various STAT family members (Hosui et al., 2017; Kotla et al., 2017; Rozovski et al., 2018).

Gene promoter translation is under the control of core binding factor (CBF) family members (Armesilla et al., 1996) and CCAAT/enhancer-binding protein α (C/EBPα) (Qiao et al., 2008). A major regulator of *CD36* expression is peroxisome proliferator activated receptor gamma (PPAR-γ), which binds to enhancer regions of *CD36* (Mikkelsen et al., 2010), but other transcription factors, such as Activating Transcription Factor 2 (ATF2), were demonstrated to induce CD36 expression as well (Raghavan et al., 2018). Genetic modifications of *CD36* have been investigated thoroughly since the identification of malaria-predisposing mutations (Aitman et al., 2000). More than 9,000 SNPs have been described in the CD36 gene, in both intronic and exonic sequences, as well as the 5′ and 3′ untranslated regions (UTRs), but only a handful of them have been associated to known pathologies (https://www.genecards.org/cgi-bin/carddisp.pl?gene=CD36#transcripts). This is due in part to the large blocks of linkage disequilibrium across the gene in several of these population studies. CD36 polymorphisms are more frequent in Asian and African American populations than Caucasians (Love-Gregory et al., 2008). Several studies have investigated CD36 polymorphism in African Americans (Love-Gregory et al., 2008; Beydoun et al., 2014) and genome wide studies comparing other ethnic groups (Coram et al., 2013; Ellis et al., 2014). A gene-centric meta-analysis of lipid traits in African, East Asian, and Hispanic populations confirmed previous data on CD36 polymorphisms and proposed the rs3211938-G allele, which is nearly absent in European and Asian populations, as a “signature of selection” in Africans and African Americans (Elbers et al., 2012).

Regarding copy number variations (CNVs), *CD36*-related modifications occur mostly with loss of genetic sequences (Vogler et al., 2010; Suktitipat et al., 2014; Uddin et al., 2015), leading to platelet glycoprotein IV deficiency or neurocognitive developmental delay (Coe et al., 2014).

In addition to genetic alterations, epigenetic and post-transcriptional interventions can also modify the final protein output, impacting protein function. Gene expression can be further altered in the cytoplasmic compartment by non-coding RNA species that can interfere with mRNA translation and modulate protein output (Pop et al., 2018). Some of these epigenetic gene control mechanisms are addressed below with respect to CD36 expression and involvement in pathology. Post-translational modifications of CD36 were reviewed recently by Luiken et al. (2016) and are beyond the scope of this review.

### Epigenetic Regulation

Epigenetics is defined as “the study of changes in gene function that are mitotically and/or meiotically heritable and that do not entail change in DNA sequence” (Wu, 2001). Although no general consensus has been reached in regard to which mechanisms can be categorized as epigenetic (Deans and Maggert, 2015), they usually involve nuclear processes, such as DNA methylation, histone modifications, and non-coding RNAs (Goldberg et al., 2007; Peschansky and Wahlestedt, 2013; Ramassone et al., 2018).

DNA methylation involves the transfer of methyl groups from S-adenosylmethionine to cytosines in CpG dinucleotide sequences. The methylation patterns of CpG sites are created and maintained by a family of DNA methyltransferases (DNMT1, DNMT3A, and DNMT3B) and can be reversed by ten-eleven translocation (TET) enzymes (Moutinho and Esteller, 2017; Pfeifer, 2018). Hypermethylation is associated with gene repression, and hypomethylation with gene activation (Morlando and Fatica, 2018). Alterations of methylation patterns play an important part in regulating gene activity during embryogenesis, gametogenesis, and cellular differentiation (Goldberg et al., 2007; Pfeifer, 2018). In many types of cancer, global hypomethylation has been observed at repetitive genomic regions, leading to genomic instability, in combination with hypermethylation at specific CpG-rich islands in the promoters of key tumor suppressor genes (Pfeifer, 2018; Morlando and Fatica, 2018; Thomas and Marcato, 2018; Ramassone et al., 2018).

Histone (H) proteins organize chromatin into structural units called nucleosomes, which consist of DNA wrapped around an octamer of H2A, H2B, H3, and H4 core histones connected by linker DNA and stabilized by H1 (Hergeth and Schneider, 2015). Epigenetic events usually include covalent modification of amino acids from the H3 and H4 tails (Cheng and Blumenthal, 2010; Iorio et al., 2010; Moutinho and Esteller, 2017). These changes represent the so-called “histone code,” which affects the conformation of the chromatin fiber, regulating the switch between euchromatin (transcriptionally active) and heterochromatin (transcriptionally inactive) (Moutinho and Esteller, 2017). Histone modifications are mediated by histone acetyltransferases (HATs), histone deacetylases (HDACs), histone methyltransferases (HMTs), and polycomb repressive complex 2 (PRC2) (Moutinho and Esteller, 2017; Thomas and Marcato, 2018). Several studies have shown an interplay between DNA methylation and histone modifications in determining the transcriptional status of a gene (Fuks et al., 2000; Johnson et al., 2002; Fuks et al., 2003; Weber et al., 2007; Cheng and Blumenthal, 2010).

Non-coding RNAs (ncRNAs) are transcribed from DNA but not translated into proteins. Although not all categories of ncRNAs can be classified as epigenetic factors, several types have been shown to control epigenetic mechanisms and, in turn, to be regulated by the epigenetic machinery (Goldberg et al., 2007; Iorio et al., 2010; Collins et al., 2011; Peschansky and Wahlestedt, 2013; Ramassone et al., 2018). The classes of ncRNAs that are epigenetically related are long non-coding RNAs (lncRNAs) and three types of short non-coding RNAs: microRNAs (miRNAs), short interfering RNAs (siRNAs), and Piwi-interacting RNAs (piRNAs) (Collins et al., 2011; Peschansky and Wahlestedt, 2013). However, the data available on CD36 regulation by ncRNAs show an involvement of the latter only in post-transcriptional mechanisms controlling CD36 mRNA levels, with no input from the epigenetic machinery.

#### DNA Methylation of *CD36*

The human CD36 gene presents several promoter sequences and distal cis-regulatory elements, which contain a number of CpG sites (Mikkelsen et al., 2010). Changes in CpG methylation patterns in the *CD36* promoters have been studied more extensively in relation to lipid metabolism and associated disorders. One study investigated the impact of *CD36* promoter SNPs and DNA methylation sites on postprandial lipid uptake and clearance in adipose and heart tissue (Love-Gregory et al., 2016). Several SNPs were associated with higher chylomicron (CM) remnants and LDL particle numbers, as well as a delayed triglyceride (TG) clearance, and some of them also correlated with lower CD36 mRNA levels and aligned to binding sites for PPAR-γ. Furthermore, the SNPs negatively related to CD36 level were associated with methylation at several CpG sites, although the two factors seem to function independently in regulating *CD36* mRNA expression (Love-Gregory et al., 2016).

Several studies have focused on obesity and its related co-morbidities: metabolic syndrome or obesity hypoventilation syndrome. In a weight loss and n-3 polyunsaturated fatty acid (PUFA) supplementation study in young overweight women, *CD36* promoter methylation was significantly reduced when adjusted for baseline body weight (Amaral et al., 2014). *CD36* was hypermethylated and less expressed in abdominal omental visceral adipose tissue (OVAT) from non-obese individuals than obese individuals (Keller et al., 2017). Fat deposits in OVAT have been shown to more strongly correlate with a higher risk of obesity-related co-morbidities than subcutaneous deposits (Vega et al., 2006). *CD36* hypermethylation has also been found in monocytes from patients with obesity hypoventilation syndrome, following the application of positive airway pressure during sleep (the current treatment for the condition) (Cortese et al., 2016). Another study investigated the methylation of the most significant TG-associated CpG in a regulatory region within *CD36* (Allum et al., 2015): expression of the main CD36 transcript in adipose tissue from obese individuals with or without metabolic syndrome was negatively associated with methylation of this regulatory region.

DNA methylation profile of *CD36* has also been investigated in liver tissue. In human primary hepatocytes, *CD36* was hypomethylated and upregulated after 5 days of valproic acid (VPA) exposure, a HDAC inhibitor, which can also modulate the expression and DNA methylation level of genes involved in liver steatosis (van Breda et al., 2018). Yu et al. (2015) reported that *CD36* hypermethylation was induced in the livers of adult offspring mice by a high-lipid, high-energy maternal diet during gestation and lactation.

In addition to metabolic diseases, CD36 can be involved in the occurrence of cancer. Only one study has focused on the DNA methylation status of *CD36* in relation to tumor progression. Sun et al. (2018) have reported a hypermethylation of *CD36*, correlated with low expression in primary lung tumors. CD36 inhibited migration, invasion, and proliferation of lung cancer cells and arrested cell cycles in G0/G1 phase. Furthermore, treatment with decitabine, an inhibitor of DNA methylation and chidamide, an HDAC inhibitor, decreased the methylation and increased the mRNA expression level of *CD36*.

Currently, most of the data regarding changes in *CD36* DNA methylation come from genome-wide or epigenome-wide studies focusing on obesity and metabolic disorders, such as diabetes. The different DNA methylation signatures found in adipose tissue from lean vs. obese individuals with or without metabolic disorders could be investigated further to establish the causality between these epigenetic variants and associated pathologies. An overlooked area of research is the involvement of *CD36* methylation in the onset of cancer.

A summary of DNA methylation changes affecting the CD36 gene is presented in **Table 1**.

**Table 1:** *CD36* epigenetic changes based on cell type/tissue.

Type of modification	Tissue/cell type	Effect	References
***DNA methylation***			
CpG methylation	Adipose and heart tissue	Decreased *CD36* mRNA	Love-Gregory et al. (2016)
	Adipose tissue	Downregulation of protein expression	Allum et al. (2015)
Hypermethylation	Omental visceral adipose tissue (non-obese individuals)	Reduced protein expression	Keller et al. (2017)
	Monocytes (obesity hypoventilation syndrome patients)	Reduced protein expression, reduced inflammation	Cortese et al. (2016)
	Hepatocytes	Upregulation, increased FA influx from peripheral tissue	van Breda et al. (2018)
	Lung tumors	Low protein expression, increased migration, proliferation and invasion of lung cancer cells	Sun et al. (2018)
***Histone methylation***			
H3K4me3 increased	Erythroid precursors CD36+ Adipocytes Monocytes	Differentiation to erythroid precursors	Cui et al. (2009); Mikkelsen et al. (2010); Bekkering et al. (2014)
H3K4me1, H3K9me1, H3K36me, H4K20me1	Erythroid precursors CD36+	Differentiation to erythroid precursors	Cui et al. (2009)
***Histone acetylation***			
H3K9Ac	Aortic macrophages subjected to chronic intermittent hypoxia	Increased *CD36* mRNA expression	Cortese et al. (2017)
H3K27Ac	Adipocytes	Open chromatin	Mikkelsen et al. (2010)
H4 acetylation	Macrophages	Increased *CD36* mRNA	Choi et al. (2005)

#### Histone Modifications of *CD36*

The CD36 gene promoters and distal enhancers that bind CCCTC-binding factor (CTCF) or PPAR-γ are subjected to both histone acetylation and methylation (Mikkelsen et al., 2010). Although histone tails can be subjected to many other covalent modifications, they have not been documented in CD36 expression. Changes in *CD36* histone marks have been studied mostly in adipocytes (Steger et al., 2008; Mikkelsen et al., 2010), monocytes/macrophages (Choi et al., 2005; Bekkering et al., 2014; Cortese et al., 2017), and hepatocytes (Cao et al., 2013; Zhong et al., 2017), but also in erythroid precursors (Cui et al., 2009), fibroblasts (Garbes et al., 2012), endothelial cells (Ren et al., 2016), and microglia (Xia et al., 2017). A summary of histone modifications affecting *CD36* is presented in **Table 1**.

Gene expression of *CD36* in human adipocytes has been shown to be activated by both H3K4me3 at P3, the major *CD36* promoter, and H3K27Ac in PPAR-γ enhancer (Mikkelsen et al., 2010). Intergenic enrichment of H3K79 monomethylation upstream of *CD36* correlates with PPAR occupancy (Steger et al., 2008). The H3K4me3 mark at the *CD36* promoter has also been linked to differentiation of hematopoietic stem/progenitor cells to erythroid precursors (Cui et al., 2009), and in macrophages it accompanies the switch to a pro-inflammatory phenotype (Bekkering et al., 2014). Another histone mark affecting CD36 expression in macrophages is H3K9Ac enrichment, which is found in aortic macrophages exposed to long-term chronic intermittent hypoxia, resulting in a higher *CD36* mRNA level (Cortese et al., 2017). Increased H3 acetylation upstream of the *CD36* promoter has also been correlated with hepatic accumulation of TGs (Cao et al., 2013).

Activation or repression of *CD36* expression *via* histone modifications can be induced pharmacologically. Using trichostatin A (TSA), a specific HDAC inhibitor, that stimulates acetylation of H4 at the *CD36* promoter, increases *CD36* mRNA, followed by higher uptake of oxLDL in macrophages (Choi et al., 2005). CD36 repression has been reported with lysophosphatidic acid, *via* HDAC7 in endothelial cells (Ren et al., 2016), and an HDAC3 inhibitor (RGFP966) in primary microglia (Xia et al., 2017).

Two studies have shown that the relationship between CD36 and histone modifiers is not unidirectional, and the former is capable of influencing the activity of the latter. Zhong et al. (2017) reported that *CD36* deletion inhibits nuclear HDAC2 expression in hepatocytes, changing the acetylation of histones binding to the monocyte chemoattractant protein-1 (MCP-1) promoters and increasing macrophage infiltration and hepatic inflammation. In a study investigating the efficiency of VPA treatment in patients with spinal muscular atrophy, non-responsiveness to the drug was linked to CD36 overexpression, which suppressed the inhibitory effect of VPA on HDACs (Garbes et al., 2012).

*CD36* histone modifications affect cells involved in lipid metabolism, but in contrast to DNA methylation patterns, changes in *CD36* histone marks in these cells have been associated mostly with inflammation (Bekkering et al., 2014; Zhong et al., 2017). Several studies activating or inhibiting CD36 *via* histone modifiers have shown the therapeutic potential of these histone marks as targets for regulating the inflammatory response (Bekkering et al., 2014; Zhong et al., 2017). Although histone modifications have been documented extensively in cancer (Audia and Campbell, 2016), no study has yet linked *CD36* histone marks with tumorigenesis.

### Post-Transcriptional Regulation

miRNAs are small single-stranded RNA molecules (18–25 nucleotides) (Sato et al., 2011; Moutinho and Esteller, 2017) that start off as long primary miRNA transcripts (pri-miRNAs), generated by RNA polymerase II. They are cleaved into hairpin precursors (pre-miRNAs) in the nucleus by a complex containing the RNAse III Drosha (Sato et al., 2011; Moutinho and Esteller, 2017), exported to the cytoplasm, and further cleaved into miRNA duplexes by RNAse III Dicer (Sato et al., 2011; Moutinho and Esteller, 2017). One of the RNA strands is incorporated into the RNA-induced silencing complex (RISC) and drives its binding to the 3′-UTR of the target mRNA, where it induces cleavage and degradation or translational repression (Collins et al., 2011; Sato et al., 2011; Moutinho and Esteller, 2017). miRNA expression can be regulated by epigenetic mechanisms (Tuna et al., 2016; Moutinho and Esteller, 2017). miRNAs themselves can control the epigenetic machinery by modulating the activity of various epigenetic effectors at a post-transcriptional level in the cytoplasm. However, miRNAs can also act in the nucleus at the transcriptional level, activating or repressing gene transcription by inducing changes in the chromatin state (Moutinho and Esteller, 2017; Ramassone et al., 2018).

lncRNAs are non-protein-coding transcripts longer than 200 nucleotides with higher tissue specificity than protein-coding genes (Morlando and Fatica, 2018). Through interaction with other molecules (proteins, DNA, other RNAs), lncRNAs can regulate gene expression at transcriptional and post-transcriptional levels and direct epigenetic changes by coordinating chromatin remodeling, affecting DNA methylation, or acting as competing endogenous RNAs by binding to the target sequence of miRNAs (Forrest and Khalil, 2017; Morlando and Fatica, 2018; Hanly et al., 2018; Hu et al., 2018).

#### CD36 Regulation by miRNAs

*CD36* mRNA is targeted by different species of miRNA that modulate its expression at a post-transcriptional level in a tissue-specific manner. **Table 2** summarizes the species of ncRNAs (miRNAs and lncRNAs) demonstrated to be involved in the regulation of CD36 expression. Zhou et al. (2016) reported that CD36 is increased during bone marrow cell differentiation towards the monocytic-macrophage line and associated with differential expression of seven miRNA species; miR-130a, -134, -141, -199a, and -363 were decreased, and miR-152 and -342-3p were increased. The miR expression profiling in erythropoiesis revealed that miR-16, miR-22, miR-26a, and miR-223 correlated with the appearance and level of CD36 as an erythroid surface antigen (Choong et al., 2007).

**Table 2:** Post-transcriptional regulation of CD36 and functional/pathological significance by cell type.

Non-coding RNA	Effect on *CD36* mRNA	Cell type	Functional/pathological significance	References
***miRNAs***				
miR-16 miR-22 miR-26a	Upregulation	Erythroid cells	CD36 erythroid surface antigen appearance	Choong et al. (2007)
miR-26a/b	Downregulation	Kidney cells (cortex)	Hypertension	Marques et al. (2011)
miR-27a	Downregulation	Pre-adipocytes	Negative regulator of adipocyte differentiation	Kim et al. (2010)
miR-27a/b	Downregulation	Macrophages	Blocking of lipid uptake	Zhang et al. (2014)
miR-29a	Upregulation	oxLDL-stimulated dendritic cells	Increased lipid uptake	Chen et al. (2011)
miR-34a	Downregulation	White adipocytes	Reduced lipid uptake	Lavery et al. (2016)
miR-130a	Downregulation	Macrophages	Macrophage differentiation	Zhou et al. (2016)
miR-133a	Downregulation	Macrophages	Reduced lipid accumulation	Peng et al. (2016)
miR-134	Downregulation	Macrophages	Macrophage differentiation	Zhou et al. (2016)
miR-135a	Downregulation	Macrophages	Inhibition of foam cell formation; decreased TG, TC levels	Du and Lu (2018)
miR-141	Downregulation	Macrophages	Macrophage differentiation	Zhou et al. (2016)
miR-152	Upregulation	Macrophages	Macrophage differentiation	Zhou et al. (2016)
miR-155	Upregulation	oxLDL-stimulated macrophages	Differentiation into dendritic cells	Ma et al. (2015)
miR-155	Downregulation	Macrophages, liver cells	Reduced lipid uptake	Federici et al. (2013)
miR-181a	Downregulation	Macrophages	Inhibition of foam cell formation; decreased TG, TC levels	Du et al. (2018)
miR-182-5p	Downregulation	Macrophages	Reduced TG, TC levels; repression of ox-LDL induced apoptosis	Qin et al. (2018)
miR-199a	Downregulation	Macrophages	Macrophage differentiation	Zhou et al. (2016)
miR-223	Upregulation	Erythroid cells	CD36 erythroid surface antigen appearance	Choong et al. (2007)
miR-342-3p	Upregulation	Macrophages	Macrophage differentiation	Zhou et al. (2016)
miR-363	Downregulation	Macrophages	Macrophage differentiation	Zhou et al. (2016)
miR-590	Downregulation	Peritoneal macrophages from apoE^−/−^ mice	Suppressed lipoprotein lipase levels	Dileepan et al. (2015)
miR-758-5p	Downregulation	Macrophages	Reduced lipid accumulation	Li et al. (2017)
***lncRNAs***				
E330013P06	Upregulation	Macrophages	Increased uptake of modified LDL, promotion of foam cell formation	Reddy et al. (2014)
MALAT1	Upregulation	Macrophages	Increased lipid uptake	Huangfu et al. (2018)
n341587, n382000	Upregulation	Peripheral blood cells of type 2 diabetes patients	Insulin resistance, hyperinsulinemia	Wang et al. (2017)
NEAT1	Downregulation	Macrophages	Reduced lipid uptake by stabilizing *CD36* mRNA in paraspeckles	Huang-Fu et al. (2017)
uc.372	Upregulation	Hepatocytes	Enhanced lipid uptake by relieving miR-4668 suppression of CD36 gene	Guo et al. (2018)

Given the association of CD36 with lipid metabolism and the robust expression on circulating monocytes and tissue macrophages, a lot of data have been collected from *in vitro* models using these cells. For example, in an *in vitro* atherosclerosis model based on oxLDL-stimulated dendritic cells derived from circulating monocytes, Chen et al. (2011) found that increased levels of miR-29a were associated with higher expression of CD36 at both the protein and mRNA level. miR-155 induced expression of CD36 in oxLDL-treated macrophages, which promoted their differentiation into dendritic cells (DCs) (So et al., 2017). In addition, silencing miR-155 in a human monocytic-macrophage cell line upregulated the expression of CD36 and increased the uptake of lipids induced by oxLDL. CD36 and scavenger receptor A (SRA) have been shown to be involved in oxLDL-mediated miR-155 upregulation in DCs (Yan et al., 2016).

Several other studies have reported post-transcriptional regulation of this fatty-acid translocator by miRNAs. miR-758-5p was shown to specifically bind to the 3′-UTR of *CD36* mRNA, downregulating both the mRNA and protein in macrophages (Li et al., 2017). Furthermore, these biochemical alterations were specifically related to reduced cellular cholesterol uptake. miR-34a is also involved in downregulation of CD36, as miR34a^−/−^ mice exhibit increased protein expression and increased lipid content in adipocytes (Lavery et al., 2016). Other miRNA species involved in downregulation of CD36 are miR-135a (Du and Lu, 2018), miR-181a (Du et al., 2018), and miR-182-5p (Qin et al., 2018).

As with histone modifiers, interactions between CD36 and miRNAs can go both ways. For example, oxLDL binding to CD36 suppresses cellular Dicer levels and subsequent miR-30c-5p expression (Ceolotto et al., 2017). In human prostate cancer cells, CD36 activation *via* TSP-2 binding downregulates miR-376c *via* a MAPK-dependent pathway (Chen et al., 2017).

Apart from several miRs that control the exposure of CD36 on the surface of erythroid precursors, most miRs species that have been shown to influence CD36 expression are involved in regulating lipid metabolism. For example, miR-133a attenuates lipid accumulation *via* the testicular orphan nuclear receptor 4 (TR4)-CD36 pathway in macrophages and may serve as a potential biomarker and potent therapeutic agent for atherosclerosis (Peng et al., 2016). On the other hand, CD36 is downregulated in hypertensive kidney (cortex), putatively targeted by miR-26a/b (Marques et al., 2011).

Despite insufficient evidence to support the clinical use of these miRs as potential diagnostic or prognostic biomarkers, further data could lead to their clinical relevance.

#### CD36 Regulation by lncRNAs

Fewer studies have investigated the roles of various lncRNAs in controlling CD36 expression, and they focus mainly on lipid uptake in macrophages. Two recent papers have demonstrated the involvement of lncRNAs metastasis-associated lung adenocarcinoma transcript 1 (MALAT1) (Huangfu et al., 2018) and nuclear-enriched abundant transcript 1 (NEAT1) (Huang-Fu et al., 2017) in oxLDL-induced CD36-mediated lipid uptake by macrophages, which plays an important role in the development of atherosclerosis. MALAT1 was first identified in non-small cell lung cancer patients (Ji et al., 2003) but has been shown to be expressed in almost all human tissues (Zhang et al., 2017). Although MALAT1 and NEAT1 loci are adjacent in the genome, their nuclear localization is distinct: MALAT1 is found in nuclear speckles, nuclear bodies that contain various pre-mRNA splicing factors, whereas NEAT1 is localized in paraspeckles, subnuclear structures present in the interchromatin space near the nuclear speckles (West et al., 2014). Huangfu et al. (2018) show that oxLDL promotes MALAT1 transcription, which induces CD36 upregulation by binding to β-catenin and promoting its accumulation on PPAR-γ responding sites of the *CD36* promoter. Even though MALAT1 is known to act in epigenetic events, such as histone modification induced by an interaction with complexes PRC1 and PRC2 (Yang et al., 2011; Abdouh et al., 2016; Qi et al., 2016; Kim et al., 2017; Biswas et al., 2018), it is not clear whether *CD36* transcription is affected by direct binding of the lncRNA to the promoter or by changing the nucleosome organization. oxLDL upregulates both NEAT1 isoforms (NEAT1 and NEAT1_2) and also stimulates NEAT1_2-mediated paraspeckle formation, which then suppresses lipid uptake by stabilizing *CD36* mRNA in paraspeckles (Huang-Fu et al., 2017).

CD36 upregulation was induced by overexpression of lncRNA E330013P06 in macrophages from diabetic mice (Reddy et al., 2014), increasing foam cell formation and contributing to an enhanced inflammatory and atherogenic phenotype in macrophages. The human equivalent of lncRNA E33, MIR143HG, which has similar genomic organization as the mouse gene, was also overexpressed in monocytes from patients with type 2 diabetes (Reddy et al., 2014). In another study focusing on aberrantly expressed lncRNAs in patients with type 2 diabetes, *CD36* mRNA expression had a high correlation coefficient with two co-expressed lncRNAs (NONCODE IDs n382000 and n341587) (Wang et al., 2017), demonstrating a possible role in the pathogenesis of type 2 diabetes through regulation of inflammation.

Finally, CD36 expression and that of other genes involved in lipid synthesis and uptake can be controlled by the interaction between an ultraconserved (uc) lncRNA and two species of miRs (Guo et al., 2018). The upregulation of uc.372 drives hepatic steatosis in mice, as well as patients with non-alcoholic fatty liver disease (NAFLD), by intervening in the maturation of miR-195 and miR-4668. In the case of *CD36*, uc.372 binds to the terminal loop region of pri-miR-4668, blocking its maturation and relieving its gene silencing effect on *CD36*. Because uc.372 promotes hepatic steatosis through these mechanisms, uc.372 inhibitors could be potential therapeutic agents for NAFLD.

## Conclusions

CD36 is a widespread protein with various functions depending on tissue localization, from adhesion protein to scavenger for oxidized phospholipids and lipoproteins, or fatty acid translocator. CD36 is involved in cardiovascular and metabolic diseases, as well as immune responses and cancer metastasis. However, CD36 has been studied in detail in some cells (monocytes-macrophages) and diseases (obesity, atherosclerosis/cardiovascular diseases), whereas other areas (tumor metastasis) are less covered. Genetic mutations in *CD36*, and changes in protein expression have been repeatedly reported in relation to cardiac artery disease, metabolic syndrome and obesity, malaria, and tumor spreading. Beyond genetic variants of *CD36*, epigenetic and post-transcriptional controls of *CD36* gene expression play a significant role in protein function and may serve as regulatory circuits, which are yet insufficiently explored. DNA methylation of *CD36* has been studied mostly in relation to lipid metabolism and obesity, whereas changes in *CD36* histone marks have been linked to inflammation. Both epigenetic mechanisms can serve as targets for drug development in their associated pathologies. Thus far, a clear relationship has been demonstrated between certain miRNAs, CD36, and altered lipid profile that can be further exploited therapeutically. However, miRNAs do not stand alone in this post-transcriptional regulation, as long-noncoding RNAs are also responsible for the stability of *CD36* mRNA. No data are yet available on the involvement of other small non-coding RNA species in CD36 expression, such as endogenous siRNAs or piRNAs. Thus, in terms of epigenetic control of CD36, there are still many gaps to be filled, especially in cancer research.

## Author Contributions

C-MN, A-ME, and MH gathered the data and wrote the manuscript; MH corrected the final proof. All authors have read and approved the submitted form.

## Funding

This manuscript was funded by the Ministry of Research and Innovation in Romania, under Program 1—The Improvement of the National System of Research and Development, Subprogram 1.2—Institutional Excellence—Projects of Excellence Funding in RDI, Contract No. 7PFE/16.10.2018 and grant COP A 1.2.3., ID: P_40_197/2016.

## Conflict of Interest Statement

The authors declare that the research was conducted in the absence of any commercial or financial relationships that could be construed as a potential conflict of interest.

## References

[B1] Abdouh M. Hanna R. El Hajjar J. Flamier A. Bernier G. (2016). The polycomb repressive complex 1 protein BMI1 is required for constitutive heterochromatin formation and silencing in mammalian somatic cells. J. Biol. Chem. 291 (1), 182–197. 10.1074/jbc.M115.662403 26468281 PMC4697155

[B2] Abumrad N. A. Maghrabi M. R. Amri E. Z. Lopez E. Grimaldi P. A. (1993). Cloning of a rat adipocyte membrane protein implicated in binding or transport of long-chain fatty acids that is induced during preadipocyte differentiation. Homology with human CD36. J. Biol. Chem. 268 (24), 17665–17668. 7688729

[B3] Aitman T. J. Cooper L. D. Norsworthy P. J. Wahid F. N. Gray J. K. Curtis B. R. (2000). Malaria susceptibility and CD36 mutation. Nature 405 (6790), 1015–1016. 10.1038/35016636 10890433

[B4] Allum F. Shao X. Guénard F. Simon M.-M. Busche S. Caron M. (2015). Characterization of functional methylomes by next-generation capture sequencing identifies novel disease-associated variants. Nat. Commun. 6, 7211. 10.1038/ncomms8211 26021296 PMC4544751

[B5] Amaral C.L.d. Milagro F. I. Curi R. Martínez J. A. (2014). DNA methylation pattern in overweight women under an energy-restricted diet supplemented with fish oil. BioMed Res. Int. 2014, 1–10. 10.1155/2014/675021 PMC3919118 24579084

[B6] Armesilla A. L. Calvo D. Vega M. A. (1996). Structural and functional characterization of the human CD36 gene promoter: identification of a proximal PEBP2/CBF site. J. Biol. Chem. 271 (13), 7781–7787. 10.1074/jbc.271.13.7781 8631821

[B7] Asch A. S. Liu I. Briccetti F. M. Barnwell J. W. Kwakye-Berko F. Dokun A. (1993). Analysis of CD36 binding domains: ligand specificity controlled by dephosphorylation of an ectodomain. Science 262 (5138), 1436–1440. 10.1126/science.7504322 7504322

[B8] Audia J. E. Campbell R. M. (2016). Histone modifications and cancer. Cold Spring Harb. Perspect. Biol. 8 (4), a019521. 10.1101/cshperspect.a019521 PMC4817802 27037415

[B9] Barnwell J. W. Asch A. S. Nachman R. L. Yamaya M. Aikawa M. Ingravallo P. (1989). A human 88-kD membrane glycoprotein (CD36) functions in vitro as a receptor for a cytoadherence ligand on Plasmodium falciparum-infected erythrocytes. J. Clin. Invest. 84 (3), 765–772. 10.1172/JCI114234 2474574 PMC329717

[B10] Bekkering S. Quintin J. Joosten L. A. B. van der Meer J. W. M. Netea M. G. Riksen N. P. (2014). Oxidized low-density lipoprotein induces long-term proinflammatory cytokine production and foam cell formation via epigenetic reprogramming of monocytes. Arterioscler. Thromb. Vasc. Biol. 34 (8), 1731–1738. 10.1161/ATVBAHA.114.303887 24903093

[B11] Beydoun M. A. Nalls M. A. Canas J. A. Evans M. K. Zonderman A. B. (2014). Gene polymorphisms and gene scores linked to low serum carotenoid status and their associations with metabolic disturbance and depressive symptoms in African-American adults. Br. J. Nutr. 112 (6), 992–1003. 10.1017/S0007114514001706 25201307 PMC4887136

[B12] Biswas S. Thomas A. A. Chen S. Aref-Eshghi E. Feng B. Gonder J. (2018). MALAT1: an epigenetic regulator of inflammation in diabetic retinopathy. Sci. Rep. 8 (1). 10.1038/s41598-018-24907-w PMC5916949 29695738

[B13] Cao Y. Xue Y. Xue L. Jiang X. Wang X. Zhang Z. (2013). Hepatic menin recruits SIRT1 to control liver steatosis through histone deacetylation. J. Hepatol. 59 (6), 1299–1306. 10.1016/j.jhep.2013.07.011 23867312

[B14] Ceolotto G. Giannella A. Albiero M. Kuppusamy M. Radu C. Simioni P. (2017). miR-30c-5p regulates macrophage-mediated inflammation and pro-atherosclerosis pathways. Cardiovasc. Res. 113 (13), 1627–1638. 10.1093/cvr/cvx157 29016810

[B15] Chen P.-C. Tang C.-H. Lin L.-W. Tsai C.-H. Chu C.-Y. Lin T.-H. (2017). Thrombospondin-2 promotes prostate cancer bone metastasis by the up-regulation of matrix metalloproteinase-2 through down-regulating miR-376c expression. J. Hematol. Oncol. 10 (1). 10.1186/s13045-017-0390-6 PMC5264454 28122633

[B16] Chen T. Li Z. Tu J. Zhu W. Ge J. Zheng X. (2011). MicroRNA-29a regulates pro-inflammatory cytokine secretion and scavenger receptor expression by targeting LPL in oxLDL-stimulated dendritic cells. FEBS Lett. 585 (4), 657–663. 10.1016/j.febslet.2011.01.027 21276447

[B17] Cheng X. Blumenthal R. M. (2010). Coordinated chromatin control: structural and functional linkage of DNA and histone methylation. Biochemistry 49 (14), 2999–3008. 10.1021/bi100213t 20210320 PMC2857722

[B18] Choi J.-H. Nam K.-H. Kim J. Baek M. W. Park J.-E. Park H.-Y. (2005). Trichostatin A exacerbates atherosclerosis in low density lipoprotein receptor-deficient mice. Arterioscler. Thromb. Vasc. Biol. 25 (11), 2404–2409. 10.1161/01.ATV.0000184758.07257.88 16141407

[B19] Choong M. L. Yang H. H. McNiece I. (2007). MicroRNA expression profiling during human cord blood-derived CD34 cell erythropoiesis. Exp. Hematol. 35 (4), 551–564. 10.1016/j.exphem.2006.12.002 17379065

[B20] Coe B. P. Witherspoon K. Rosenfeld J. A. van Bon B. W. Vulto-van Silfhout A. T. Bosco P. (2014). Refining analyses of copy number variation identifies specific genes associated with developmental delay. Nat. Genet. 46 (10), 1063–1071. 10.1038/ng.3092 25217958 PMC4177294

[B21] Collins L. J. Schönfeld B. Chen X. S. (2011). The epigenetics of non-coding RNA. 49–61. 10.1016/B978-0-12-375709-8.00004-6

[B22] Coram M. A. Duan Q. Hoffmann T. J. Thornton T. Knowles J. W. Johnson N. A. (2013). Genome-wide characterization of shared and distinct genetic components that influence blood lipid levels in ethnically diverse human populations. Am. J. Hum. Genet. 92 (6), 904–916. 10.1016/j.ajhg.2013.04.025 23726366 PMC3675231

[B23] Corpeleijn E. Pelsers M. M. Soenen S. Mensink M. Bouwman F. G. Kooi M. E. (2008). Insulin acutely upregulates protein expression of the fatty acid transporter CD36 in human skeletal muscle in vivo. J. Physiol. Pharmacol. 59 (1), 77–83. 18441389

[B24] Cortese R. Gileles-Hillel A. Khalyfa A. Almendros I. Akbarpour M. Khalyfa A. A. (2017). Aorta macrophage inflammatory and epigenetic changes in a murine model of obstructive sleep apnea: potential role of CD36. Sci. Rep. 7 (1). 10.1038/srep43648 PMC5327416 28240319

[B25] Cortese R. Zhang C. Bao R. Andrade J. Khalyfa A. Mokhlesi B. (2016). DNA methylation profiling of blood monocytes in patients with obesity hypoventilation syndrome. Chest 150 (1), 91–101. 10.1016/j.chest.2016.02.648 26923628 PMC6026248

[B26] Cui K. Zang C. Roh T.-Y. Schones D. E. Childs R. W. Peng W. (2009). Chromatin signatures in multipotent human hematopoietic stem cells indicate the fate of bivalent genes during differentiation. Cell Stem Cell 4 (1), 80–93. 10.1016/j.stem.2008.11.011 19128795 PMC2785912

[B27] Deans C. Maggert K. A. (2015). What do you mean, “epigenetic”? Genetics 199 (4), 887–896. 10.1534/genetics.114.173492 25855649 PMC4391566

[B28] Dileepan K. N. He P.-P. OuYang X.-P. Li Y. Lv Y.-C. Wang Z.-B. (2015). MicroRNA-590 inhibits lipoprotein lipase expression and prevents atherosclerosis in apoE knockout mice. PLoS One 10 (9), e0138788. 10.1371/journal.pone.0138788 26397958 PMC4580638

[B29] Du X.-J. Lu J.-M. (2018). MiR-135a represses oxidative stress and vascular inflammatory events via targeting toll-like receptor 4 in atherogenesis. J. Cell. Biochem. 119 (7), 6154–6161. 10.1002/jcb.26819 29663503

[B30] Du X.-J. Lu J.-M. Sha Y. (2018). MiR-181a inhibits vascular inflammation induced by ox-LDL via targeting TLR4 in human macrophages. J. Cell. Physiol. 233 (10), 6996–7003. 10.1002/jcp.26622 29737518

[B31] Elbers C. C. Guo Y. Tragante V. van Iperen E. P. Lanktree M. B. Castillo B. A. (2012). Gene-centric meta-analysis of lipid traits in African, East Asian and Hispanic populations. PLoS One 7 (12), e50198. 10.1371/journal.pone.0050198 23236364 PMC3517599

[B32] Ellis J. Lange E. M. Li J. Dupuis J. Baumert J. Walston J. D. (2014). Large multiethnic candidate gene study for C-reactive protein levels: identification of a novel association at CD36 in African Americans. Hum. Genet. 133 (8), 985–995. 10.1007/s00439-014-1439-z 24643644 PMC4104766

[B33] Enciu A. M. Radu E. Popescu I. D. Hinescu M. E. Ceafalan L. C. (2018). Targeting CD36 as biomarker for metastasis prognostic: how far from translation into clinical practice? BioMed Res. Int. 2018, 7801202. 10.1155/2018/7801202 30069479 PMC6057354

[B34] Endemann G. Stanton L. W. Madden K. S. Bryant C. M. White R. T. Protter A. A. (1993). CD36 is a receptor for oxidized low density lipoprotein. J. Biol. Chem. 268 (16), 11811–11816. 7685021

[B35] Febbraio M. Hajjar D. P. Silverstein R. L. (2001). CD36: a class B scavenger receptor involved in angiogenesis, atherosclerosis, inflammation, and lipid metabolism. J. Clin. Invest. 108 (6), 785–791. 10.1172/JCI14006 11560944 PMC200943

[B36] Federici M. Miller A. M. Gilchrist D. S. Nijjar J. Araldi E. Ramirez C. M. (2013). MiR-155 has a protective role in the development of non-alcoholic hepatosteatosis in mice. PLoS One 8 (8), e72324. 10.1371/journal.pone.0072324 23991091 PMC3749101

[B37] Forrest M. E. Khalil A. M. (2017). Review: regulation of the cancer epigenome by long non-coding RNAs. Cancer Lett. 407, 106–112. 10.1016/j.canlet.2017.03.040 28400335

[B38] Fuks F. Burgers W. A. Brehm A. Hughes-Davies L. Kouzarides T. (2000). DNA methyltransferase Dnmt1 associates with histone deacetylase activity. Nat. Genet. 24 (1), 88–91. 10.1038/71750 10615135

[B39] Fuks F. Hurd P. J. Wolf D. Nan X. Bird A. P. Kouzarides T. (2003). The methyl-CpG-binding protein MeCP2 links DNA methylation to histone methylation. J. Biol. Chem. 278 (6), 4035–4040. 10.1074/jbc.M210256200 12427740

[B40] Garbes L. Heesen L. Holker I. Bauer T. Schreml J. Zimmermann K. (2012). VPA response in SMA is suppressed by the fatty acid translocase CD36. Hum. Mol. Genet. 22 (2), 398–407. 10.1093/hmg/dds437 23077215

[B41] Glatz J. F. Luiken J. J. (2017). From fat to FAT (CD36/SR-B2): understanding the regulation of cellular fatty acid uptake. Biochimie 136, 21–26. 10.1016/j.biochi.2016.12.007 28013071

[B42] Goldberg A. D. Allis C. D. Bernstein E. (2007). Epigenetics: a landscape takes shape. Cell 128 (4), 635–638. 10.1016/j.cell.2007.02.006 17320500

[B43] Goyenechea E. Collins L. J. Parra D. Liu G. Snieder H. Swaminathan R. (2008). CD36 gene promoter polymorphisms are associated with low density lipoprotein-cholesterol in normal twins and after a low-calorie diet in obese subjects. Twin Res. Hum. Genet. 11 (6), 621–628. 10.1375/twin.11.6.621 19016618

[B44] Guo J. Fang W. Sun L. Lu Y. Dou L. Huang X. (2018). Ultraconserved element uc.372 drives hepatic lipid accumulation by suppressing miR-195/miR4668 maturation. Nat. Commun. 9, 612. 10.1038/s41467-018-03072-8 29426937 PMC5807361

[B45] Hanly D. J. Esteller M. Berdasco M. (2018). Interplay between long non-coding RNAs and epigenetic machinery: emerging targets in cancer? Philos. Trans. R Soc. Lond. B Biol. Sci. 373 (1748), 20170074. 10.1098/rstb.2017.0074 29685978 PMC5915718

[B46] Hergeth S. P. Schneider R. (2015). The H1 linker histones: multifunctional proteins beyond the nucleosomal core particle. EMBO Rep. 16 (11), 1439–1453. 10.15252/embr.201540749 26474902 PMC4641498

[B47] Hosui A. Tatsumi T. Hikita H. Saito Y. Hiramatsu N. Tsujii M. (2017). Signal transducer and activator of transcription 5 plays a crucial role in hepatic lipid metabolism through regulation of CD36 expression. Hepatol. Res. 47 (8), 813–825. 10.1111/hepr.12816 27593674

[B48] Hu G. Niu F. Humburg B. A. Liao K. Bendi V. S. Callen S. (2018). Molecular mechanisms of long noncoding RNAs and their role in disease pathogenesis. Oncotarget 9 (26). 10.18632/oncotarget.24307 PMC5915100 29719633

[B49] Huang-Fu N. Cheng J. S. Wang Y. Li Z. W. Wang S. H. (2017). Neat1 regulates oxidized low-density lipoprotein-induced inflammation and lipid uptake in macrophages via paraspeckle formation. Mol. Med. Rep. 10.3892/mmr.2017.8211 PMC5783531 29257236

[B50] Huangfu N. Xu Z. Zheng W. Wang Y. Cheng J. Chen X. (2018). LncRNA MALAT1 regulates oxLDL-induced CD36 expression via activating β-catenin. Biochem. Biophys. Res. Commun. 495 (3), 2111–2117. 10.1016/j.bbrc.2017.12.086 29258822

[B51] Iorio M. V. Piovan C. Croce C. M. (2010). Interplay between microRNAs and the epigenetic machinery: an intricate network. Biochim. Biophys. Acta Gene Regul. Mech. 1799 (10–12), 694–701. 10.1016/j.bbagrm.2010.05.005 20493980

[B52] Jay A. G. Hamilton J. A. (2018). The enigmatic membrane fatty acid transporter CD36: new insights into fatty acid binding and their effects on uptake of oxidized LDL. Prostaglandins Leukot. Essent. Fatty Acids 138, 64–70. 10.1016/j.plefa.2016.05.005 27288302

[B53] Ji P. Diederichs S. Wang W. Böing S. Metzger R. Schneider P. M. (2003). MALAT-1, a novel noncoding RNA and thymosin β4 predict metastasis and survival in early-stage non-small cell lung cancer. Oncogene 22 (39), 8031–8041. 10.1038/sj.onc.1206928 12970751

[B54] Johnson L. M. Cao X. Jacobsen S. E. (2002). Interplay between two epigenetic marks. Curr. Biol. 12 (16), 1360–1367. 10.1016/S0960-9822(02)00976-4 12194816

[B55] Keller K. L. Liang L. C. Sakimura J. May D. van Belle C. Breen C. (2012). Common variants in the CD36 gene are associated with oral fat perception, fat preferences, and obesity in African Americans. Obesity (Silver Spring) 20 (5), 1066–1073. 10.1038/oby.2011.374 22240721 PMC3743670

[B56] Keller M. Hopp L. Liu X. Wohland T. Rohde K. Cancello R. (2017). Genome-wide DNA promoter methylation and transcriptome analysis in human adipose tissue unravels novel candidate genes for obesity. Mol. Metab. 6 (1), 86–100. 10.1016/j.molmet.2016.11.003 28123940 PMC5220399

[B57] Kim S. H. Kim S. H. Yang W. I. Kim S. J. Yoon S. O. (2017). Association of the long non-coding RNA MALAT1 with the polycomb repressive complex pathway in T and NK cell lymphoma. Oncotarget 8 (19). 10.18632/oncotarget.15453 PMC5458209 28412742

[B58] Kim S. Y. Kim A. Y. Lee H. W. Son Y. H. Lee G. Y. Lee J.-W. (2010). miR-27a is a negative regulator of adipocyte differentiation via suppressing PPARγ expression. Biochem. Biophys. Res. Commun. 392 (3), 323–328. 10.1016/j.bbrc.2010.01.012 20060380

[B59] Klenotic P. A. Page R. C. Li W. Amick J. Misra S. Silverstein R. L. (2013). Molecular basis of antiangiogenic thrombospondin-1 type 1 repeat domain interactions with CD36. Arterioscler. Thromb. Vasc. Biol. 33 (7), 1655–1662. 10.1161/ATVBAHA.113.301523 23640500 PMC3737738

[B60] Kotla S. Singh N. K. Rao G. N. (2017). ROS via BTK-p300-STAT1-PPARgamma signaling activation mediates cholesterol crystals-induced CD36 expression and foam cell formation. Redox Biol. 11, 350–364. 10.1016/j.redox.2016.12.005 28040583 PMC5200884

[B61] Ladanyi A. Mukherjee A. Kenny H. A. Johnson A. Mitra A. K. Sundaresan S. (2018). Adipocyte-induced CD36 expression drives ovarian cancer progression and metastasis. Oncogene 37 (17), 2285–2301. 10.1038/s41388-017-0093-z 29398710 PMC5920730

[B62] Laugerette F. Passilly-Degrace P. Patris B. Niot I. Febbraio M. Montmayeur J. P. (2005). CD36 involvement in orosensory detection of dietary lipids, spontaneous fat preference, and digestive secretions. J. Clin. Invest. 115 (11), 3177–3184. 10.1172/JCI25299 16276419 PMC1265871

[B63] Lavery C. A. Kurowska-Stolarska M. Holmes W. M. Donnelly I. Caslake M. Collier A. (2016). miR-34a–/– mice are susceptible to diet-induced obesity. Obesity 24 (8), 1741–1751. 10.1002/oby.21561 27377585 PMC4979678

[B64] Li B.-R. Xia L.-Q. Liu J. liao L.-L. Zhang Y. Deng M. (2017). miR-758-5p regulates cholesterol uptake via targeting the CD36 3′UTR. Biochem. Biophys. Res. Commun. 494 (1–2), 384–389. 10.1016/j.bbrc.2017.09.150 28965954

[B65] Li W. Febbraio M. Reddy S. P. Yu D.-Y. Yamamoto M. Silverstein R. L. (2010). CD36 participates in a signaling pathway that regulates ROS formation in murine VSMCs. J. Clin. Invest. 120 (11), 3996–4006. 10.1172/JCI42823 20978343 PMC2964976

[B66] Liang Y. Han H. Liu L. Duan Y. Yang X. Ma C. (2018). CD36 plays a critical role in proliferation, migration and tamoxifen-inhibited growth of ER-positive breast cancer cells. Oncogenesis 7 (12), 98. 10.1038/s41389-018-0107-x 30573731 PMC6302092

[B67] Love-Gregory L. Kraja A. T. Allum F. Aslibekyan S. Hedman A. K. Duan Y. (2016). Higher chylomicron remnants and LDL particle numbers associate with CD36 SNPs and DNA methylation sites that reduce CD36. J. Lipid Res. 57 (12), 2176–2184. 10.1194/jlr.P065250 27729386 PMC5321222

[B68] Love-Gregory L. Sherva R. Sun L. Wasson J. Schappe T. Doria A. (2008). Variants in the CD36 gene associate with the metabolic syndrome and high-density lipoprotein cholesterol. Hum. Mol. Genet. 17 (11), 1695–1704. 10.1093/hmg/ddn060 18305138 PMC2655228

[B69] Luiken J. J. Chanda D. Nabben M. Neumann D. Glatz J. F. (2016). Post-translational modifications of CD36 (SR-B2): implications for regulation of myocellular fatty acid uptake. Biochim. Biophys. Acta 1862 (12), 2253–2258. 10.1016/j.bbadis.2016.09.004 27615427

[B70] Ma Y. L. Ma Z. J. Wang M. Liao M. Y. Yao R. Liao Y. H. (2015). MicroRNA-155 induces differentiation of RAW264.7 cells into dendritic-like cells. Int. J. Clin. Exp. Pathol. 8 (11), 14050–14062. 26823719 PMC4713505

[B71] Marques F. Z. Campain A. E. Tomaszewski M. Zukowska-Szczechowska E. Yang Y. H. J. Charchar F. J. (2011). Gene expression profiling reveals renin mRNA overexpression in human hypertensive kidneys and a role for microRNAs. Hypertension 58 (6), 1093–1098. 10.1161/HYPERTENSIONAHA.111.180729 22042811

[B72] Mikkelsen T. S. Xu Z. Zhang X. Wang L. Gimble J. M. Lander E. S. (2010). Comparative epigenomic analysis of murine and human adipogenesis. Cell 143 (1), 156–169. 10.1016/j.cell.2010.09.006 20887899 PMC2950833

[B73] Morlando M. Fatica A. (2018). Alteration of epigenetic regulation by long noncoding RNAs in cancer. Int. J. Mol. Sci. 19 (2), 570. 10.3390/ijms19020570 PMC5855792 29443889

[B74] Moutinho C. Esteller M. (2017). MicroRNAs and epigenetics. Adv. Cancer Res. 135, 189–220. 10.1016/bs.acr.2017.06.003 28882223

[B75] Oberland S. Ackels T. Gaab S. Pelz T. Spehr J. Spehr M. (2015). CD36 is involved in oleic acid detection by the murine olfactory system. Front. Cell Neurosci. 9, 366. 10.3389/fncel.2015.00366 26441537 PMC4584952

[B76] Ozdener M. H. Subramaniam S. Sundaresan S. Sery O. Hashimoto T. Asakawa Y. (2014). CD36- and GPR120-mediated Ca²^+^ signaling in human taste bud cells mediates differential responses to fatty acids and is -altered in obese mice. Gastroenterology 146 (4), 995–1005. 10.1053/j.gastro.2014.01.006 24412488 PMC3979457

[B77] Pan J. Fan Z. Wang Z. Dai Q. Xiang Z. Yuan F. (2019). CD36 mediates palmitate acid-induced metastasis of gastric cancer via AKT/GSK-3beta/beta-catenin pathway. J. Exp. Clin. Cancer Res. 38 (1), 52. 10.1186/s13046-019-1049-7 30717785 PMC6360779

[B78] Park Y. M. (2014). CD36, a scavenger receptor implicated in atherosclerosis. Exp. Mol. Med. 46, e99. 10.1038/emm.2014.38 24903227 PMC4081553

[B79] Pascual G. Avgustinova A. Mejetta S. Martin M. Castellanos A. Attolini C. S. (2017). Targeting metastasis-initiating cells through the fatty acid receptor CD36. Nature 541 (7635), 41–45. 10.1038/nature20791 27974793

[B80] Peng X.-P. Huang L. Liu Z.-H. (2016). miRNA-133a attenuates lipid accumulation via TR4-CD36 pathway in macrophages. Biochimie 127, 79–85. 10.1016/j.biochi.2016.04.012 27109382

[B81] Pepino M. Y. Love-Gregory L. Klein S. Abumrad N. A. (2012). The fatty acid translocase gene CD36 and lingual lipase influence oral sensitivity to fat in obese subjects. J. Lipid Res. 53 (3), 561–566. 10.1194/jlr.M021873 22210925 PMC3276480

[B82] Peschansky V. J. Wahlestedt C. (2013). Non-coding RNAs as direct and indirect modulators of epigenetic regulation. Epigenetics 9 (1), 3–12. 10.4161/epi.27473 PMC3928183 24739571

[B83] Pfeifer G. (2018). Defining driver DNA methylation changes in human cancer. Int. J. Mol. Sci. 19 (4), 1166. 10.3390/ijms19041166 PMC5979276 29649096

[B84] Pietka T. A. Schappe T. Conte C. Fabbrini E. Patterson B. W. Klein S. (2014). Adipose and muscle tissue profile of CD36 transcripts in obese subjects highlights the role of CD36 in fatty acid homeostasis and insulin resistance. Diabetes Care 37 (7), 1990–1997. 10.2337/dc13-2835 24784828 PMC4067395

[B85] Pop S. Enciu A. M. Necula L. G. Tanase C. (2018). Long non-coding RNAs in brain tumours: focus on recent epigenetic findings in glioma. J. Cell. Mol. Med. 22 (10), 4597–4610. 10.1111/jcmm.13781 30117678 PMC6156469

[B86] Portal V. L. Markoski M. M. Quadros A. S. Garofallo S. Santos J. L. Oliveira A. (2016). Effect of polymorphisms in the CD36 and STAT3 genes on different dietary interventions among patients with coronary artery disease: study protocol for a randomized controlled trial. Trials 17 (1), 437. 10.1186/s13063-016-1564-1 27596284 PMC5011844

[B87] Qi Y. Ooi H. S. Wu J. Chen J. Zhang X. Tan S. (2016). MALAT1 long ncRNA promotes gastric cancer metastasis by suppressing PCDH10. Oncotarget 7 (11). 10.18632/oncotarget.7281 PMC4914315 26871474

[B88] Qiao L. Zou C. Shao P. Schaack J. Johnson P. F. Shao J. (2008). Transcriptional regulation of fatty acid translocase/CD36 expression by CCAAT/enhancer-binding protein α. J. Biol. Chem. 283 (14), 8788–8795. 10.1074/jbc.M800055200 18263877 PMC2276366

[B89] Qin M. Wang L. Li F. Yang M. Song L. Tian F. (2017). Oxidized LDL activated eosinophil polarize macrophage phenotype from M2 to M1 through activation of CD36 scavenger receptor. Atherosclerosis 263, 82–91. 10.1016/j.atherosclerosis.2017.05.011 28623741 PMC5557672

[B90] Qin S.-B. Peng D.-Y. Lu J.-M. Ke Z.-P. (2018). MiR-182-5p inhibited oxidative stress and apoptosis triggered by oxidized low-density lipoprotein via targeting toll-like receptor 4. J. Cell. Physiol. 233 (10), 6630–6637. 10.1002/jcp.26389 29226948

[B91] Raghavan S. Singh N. K. Gali S. Mani A. M. Rao G. N. (2018). Protein kinase Cθ via activating transcription factor 2-mediated CD36 expression and foam cell formation of Ly6Chi cells contributes to atherosclerosis. Circulation 138 (21), 2395–2412. 10.1161/CIRCULATIONAHA.118.034083 29991487 PMC6309268

[B92] Ramassone A. Pagotto S. Veronese A. Visone R. (2018). Epigenetics and microRNAs in cancer. Int. J. Mol. Sci. 19 (2), 459. 10.3390/ijms19020459 PMC5855681 29401683

[B93] Reddy M. A. Chen Z. Park J. T. Wang M. Lanting L. Zhang Q. (2014). Regulation of inflammatory phenotype in macrophages by a diabetes-induced long noncoding RNA. Diabetes 63 (12), 4249–4261. 10.2337/db14-0298 25008173 PMC4238007

[B94] Ren B. Best B. Ramakrishnan D. P. Walcott B. P. Storz P. Silverstein R. L. (2016). LPA/PKD-1-FoxO1 signaling axis mediates endothelial cell CD36 transcriptional repression and proangiogenic and proarteriogenic reprogramming. Arterioscler. Thromb. Vasc. Biol. 36 (6), 1197–1208. 10.1161/ATVBAHA.116.307421 27013613 PMC4882231

[B95] Rigotti A. Acton S. L. Krieger M. (1995). The class B scavenger receptors SR-BI and CD36 are receptors for anionic phospholipids. J. Biol. Chem. 270 (27), 16221–16224. 10.1074/jbc.270.27.16221 7541795

[B96] Rozovski U. Harris D. M. Li P. Liu Z. Jain P. Ferrajoli A. (2018). STAT3-activated CD36 facilitates fatty acid uptake in chronic lymphocytic leukemia cells. Oncotarget 9 (30), 21268–21280. 10.18632/oncotarget.25066 29765537 PMC5940394

[B97] Sato F. Tsuchiya S. Meltzer S. J. Shimizu K. (2011). MicroRNAs and epigenetics. FEBS J. 278 (10), 1598–1609. 10.1111/j.1742-4658.2011.08089.x 21395977

[B98] Silverstein R. L. Febbraio M. (2009). CD36, a scavenger receptor involved in immunity, metabolism, angiogenesis, and behavior. Sci. Signal. 2 (72), re3–re3. 10.1126/scisignal.272re3 PMC2811062 19471024

[B99] Smith J. Su X. El-Maghrabi R. Stahl P. D. Abumrad N. A. (2008). Opposite regulation of CD36 ubiquitination by fatty acids and insulin: effects on fatty acid uptake. J. Biol. Chem. 283 (20), 13578–13585. 10.1074/jbc.M800008200 18353783 PMC2376227

[B100] So E. Y. Winchester T. Ouchi T. (2017). The screening of a microRNA expression during development of human macrophages and mouse dendritic cells. Cancer Biol. Ther. 18 (3), 152–157. 10.1080/15384047.2017.1281498 28296555 PMC5389418

[B101] Sp N. Kang D. Y. Kim D. H. Park J. H. Lee H. G. Kim H. J. (2018). Nobiletin inhibits CD36-dependent tumor angiogenesis, migration, invasion, and sphere formation through the Cd36/Stat3/Nf-Kappab signaling axis. Nutrients 10 (6), E772. 10.3390/nu10060772 29914089 PMC6024609

[B102] Steger D. J. Lefterova M. I. Ying L. Stonestrom A. J. Schupp M. Zhuo D. (2008). DOT1L/KMT4 recruitment and H3K79 methylation are ubiquitously coupled with gene transcription in mammalian cells. Mol. Cell. Biol. 28 (8), 2825–2839. 10.1128/MCB.02076-07 18285465 PMC2293113

[B103] Stewart C. R. Stuart L. M. Wilkinson K. van Gils J. M. Deng J. Halle A. (2010). CD36 ligands promote sterile inflammation through assembly of a Toll-like receptor 4 and 6 heterodimer. Nat. Immunol. 11 (2), 155–161. 10.1038/ni.1836 20037584 PMC2809046

[B104] Suktitipat B. Naktang C. Mhuantong W. Tularak T. Artiwet P. Pasomsap E. (2014). Copy number variation in Thai population. PLoS One 9 (8), e104355. 10.1371/journal.pone.0104355 25118596 PMC4131886

[B105] Sun Q. Zhang W. Wang L. Guo F. Song D. Zhang Q. (2018). Hypermethylated CD36 gene affected the progression of lung cancer. Gene 678, 395–406. 10.1016/j.gene.2018.06.101 29969695

[B106] Tandon N. N. Kralisz U. Jamieson G. A. (1989). Identification of glycoprotein IV (CD36) as a primary receptor for platelet-collagen adhesion. J. Biol. Chem. 264 (13), 7576–7583. 2468670

[B107] Thomas M. Marcato P. (2018). Epigenetic modifications as biomarkers of tumor development, therapy response, and recurrence across the cancer care continuum. Cancers 10 (4), 101. 10.3390/cancers10040101 PMC5923356 29614786

[B108] Thylur R. P. Wu X. Gowda N. M. Punnath K. Neelgund S. E. Febbraio M. (2017). CD36 receptor regulates malaria-induced immune responses primarily at early blood stage infection contributing to parasitemia control and resistance to mortality. J. Biol. Chem. 292 (22), 9394–9408. 10.1074/jbc.M117.781294 28416609 PMC5454118

[B109] Tran T. T. Poirier H. Clement L. Nassir F. Pelsers M. M. Petit V. (2011). Luminal lipid regulates CD36 levels and downstream signaling to stimulate chylomicron synthesis. J. Biol. Chem. 286 (28), 25201–25210. 10.1074/jbc.M111.233551 21610069 PMC3137091

[B110] Tuna M. Machado A. S. Calin G. A. (2016). Genetic and epigenetic alterations of microRNAs and implications for human cancers and other diseases. Genes, Chromosomes and Cancer 55 (3), 193–214. 10.1002/gcc.22332 26651018

[B111] Uddin M. Thiruvahindrapuram B. Walker S. Wang Z. Hu P. Lamoureux S. (2015). A high-resolution copy-number variation resource for clinical and population genetics. Genet. Med. 17 (9), 747–752. 10.1038/gim.2014.178 25503493 PMC4752593

[B112] van Breda S. G. J. Claessen S. M. H. van Herwijnen M. Theunissen D. H. J. Jennen D. G. J. de Kok T. M. C. M. (2018). Integrative omics data analyses of repeated dose toxicity of valproic acid in vitro reveal new mechanisms of steatosis induction. Toxicology 393, 160–170. 10.1016/j.tox.2017.11.013 29154799

[B113] Vega G. L. Adams-Huet B. Peshock R. Willett D. Shah B. Grundy S. M. (2006). Influence of body fat content and distribution on variation in metabolic risk. J. Clin. Endocrinol. Metab. 91 (11), 4459–4466. 10.1210/jc.2006-0814 16926254

[B114] Vogler C. Gschwind L. Rothlisberger B. Huber A. Filges I. Miny P. (2010). Microarray-based maps of copy-number variant regions in European and sub-Saharan populations. PLoS One 5 (12), e15246. 10.1371/journal.pone.0015246 21179565 PMC3002949

[B115] Wang X. Chang X. Zhang P. Fan L. Zhou T. Sun K. (2017). Aberrant expression of long non-coding RNAs in newly diagnosed type 2 diabetes indicates potential roles in chronic inflammation and insulin resistance. Cell. Physiol. Biochem. 43 (6), 2367–2378. 10.1159/000484388 29073614

[B116] Weber M. Hellmann I. Stadler M. B. Ramos L. Pääbo S. Rebhan M. (2007). Distribution, silencing potential and evolutionary impact of promoter DNA methylation in the human genome. Nat. Genet. 39 (4), 457–466. 10.1038/ng1990 17334365

[B117] West J. A. Davis C. P. Sunwoo H. Simon M. D. Sadreyev R. I. Wang P. I. (2014). The long noncoding RNAs NEAT1 and MALAT1 bind active chromatin sites. Mol. Cell 55 (5), 791–802. 10.1016/j.molcel.2014.07.012 25155612 PMC4428586

[B118] Wu, C.t. (2001). Genes, genetics, and epigenetics: a correspondence. Science 293 (5532), 1103–1105. 10.1126/science.293.5532.1103 11498582

[B119] Xavier A. M. Ludwig R. G. Nagai M. H. de Almeida T. J. Watanabe H. M. Hirata M. Y. (2016). CD36 is expressed in a defined subpopulation of neurons in the olfactory epithelium. Sci Rep 6, 25507. 10.1038/srep25507 27145700 PMC4857125

[B120] Xia M. Zhao Q. Zhang H. Chen Y. Yuan Z. Xu Y. (2017). Proteomic analysis of HDAC3 selective inhibitor in the regulation of inflammatory response of primary microglia. Neural Plast. 2017, 1–13. 10.1155/2017/6237351 PMC5331322 28293439

[B121] Yan H. U. I. Wang S. Li Z. Zhao W. Wang Z. Sun Z. (2016). Upregulation of miRNA-155 expression by OxLDL in dendritic cells involves JAK1/2 kinase and transcription factors YY1 and MYB. Int. J. Mol. Med. 37 (5), 1371–1378. 10.3892/ijmm.2016.2526 26985867

[B122] Yang L. Lin C. Liu W. Zhang J. Ohgi K. A. Grinstein J. D. (2011). ncRNA- and Pc2 methylation-dependent gene relocation between nuclear structures mediates gene activation programs. Cell 147 (4), 773–788. 10.1016/j.cell.2011.08.054 22078878 PMC3297197

[B123] Yu H.-L. Dong S. Gao L.-F. Li L. Xi Y.-D. Ma W.-W. (2015). Global DNA methylation was changed by a maternal high-lipid, high-energy diet during gestation and lactation in male adult mice liver. Br. J. Nutr. 113 (07), 1032–1039. 10.1017/S0007114515000252 25778733

[B124] Yue H. Febbraio M. Klenotic P. A. Kennedy D. J. Wu Y. Chen S. (2019). CD36 enhances vascular smooth muscle cell proliferation and development of neointimal hyperplasia. Arterioscler. Thromb. Vasc. Biol. 39 (2), 263–275. 10.1161/ATVBAHA.118.312186 30567481 PMC6345504

[B125] Zhang M. Wu J.-F. Chen W.-J. Tang S.-L. Mo Z.-C. Tang Y.-Y. (2014). MicroRNA-27a/b regulates cellular cholesterol efflux, influx and esterification/hydrolysis in THP-1 macrophages. Atherosclerosis 234 (1), 54–64. 10.1016/j.atherosclerosis.2014.02.008 24608080

[B126] Zhang X. Hamblin M. H. Yin K.-J. (2017). The long noncoding RNA Malat1: its physiological and pathophysiological functions. RNA Biol. 14 (12), 1705–1714. 10.1080/15476286.2017.1358347 28837398 PMC5731810

[B127] Zhong S. Zhao L. Wang Y. Zhang C. Liu J. Wang P. (2017). Cluster of differentiation 36 deficiency aggravates macrophage infiltration and hepatic inflammation by upregulating monocyte chemotactic protein-1 expression of hepatocytes through histone deacetylase 2-dependent pathway. Antioxid. Redox Signal. 27 (4), 201–214. 10.1089/ars.2016.6808 27967209

[B128] Zhou H. Zhang J. Eyers F. Xiang Y. Herbert C. Tay H. L. (2016). Identification of the microRNA networks contributing to macrophage differentiation and function. Oncotarget 7 (20), 28806–28820. 10.18632/oncotarget.8933 27119502 PMC5045358

